# Hernia of Urinary Bladder

**Published:** 1915-03

**Authors:** 


**Hernia of Urinary Bladder.** W. O. Roberts, Louisville, Louisville Mo. Jour. of Med. & Surg., Feb., 1915. This condition was recognized by Albucasis in the eleventh century. In 1908 about 300 cases had been collected and since then, they are reported at the rate of about one a month. The author mentions 2 personal cases. It is mentioned as a complication in 23 cases in about 2500 hernia operations by 8 surgeons. O. R. Miller of the N. Y. Hospital for Ruptured and Crippled, has encountered the condition 8 times in 4,285 records; 5 times in 3,119 male children; never in female children; in adult females, once in 259 cases of indirect inguinal hernia, once in 7 cases of direct inguinal, one in 181 cases of femoral hernia. (Male adults not taken at this hospital).

